# Supplementary figures and images for: The Functional Significance of Bacterial Predators
Source: mBio. 2021 Apr 27;12(2):e00466-21. doi: 10.1128/mBio.00466-21 (PMC8092244; doi:10.1128/mBio.00466-21)

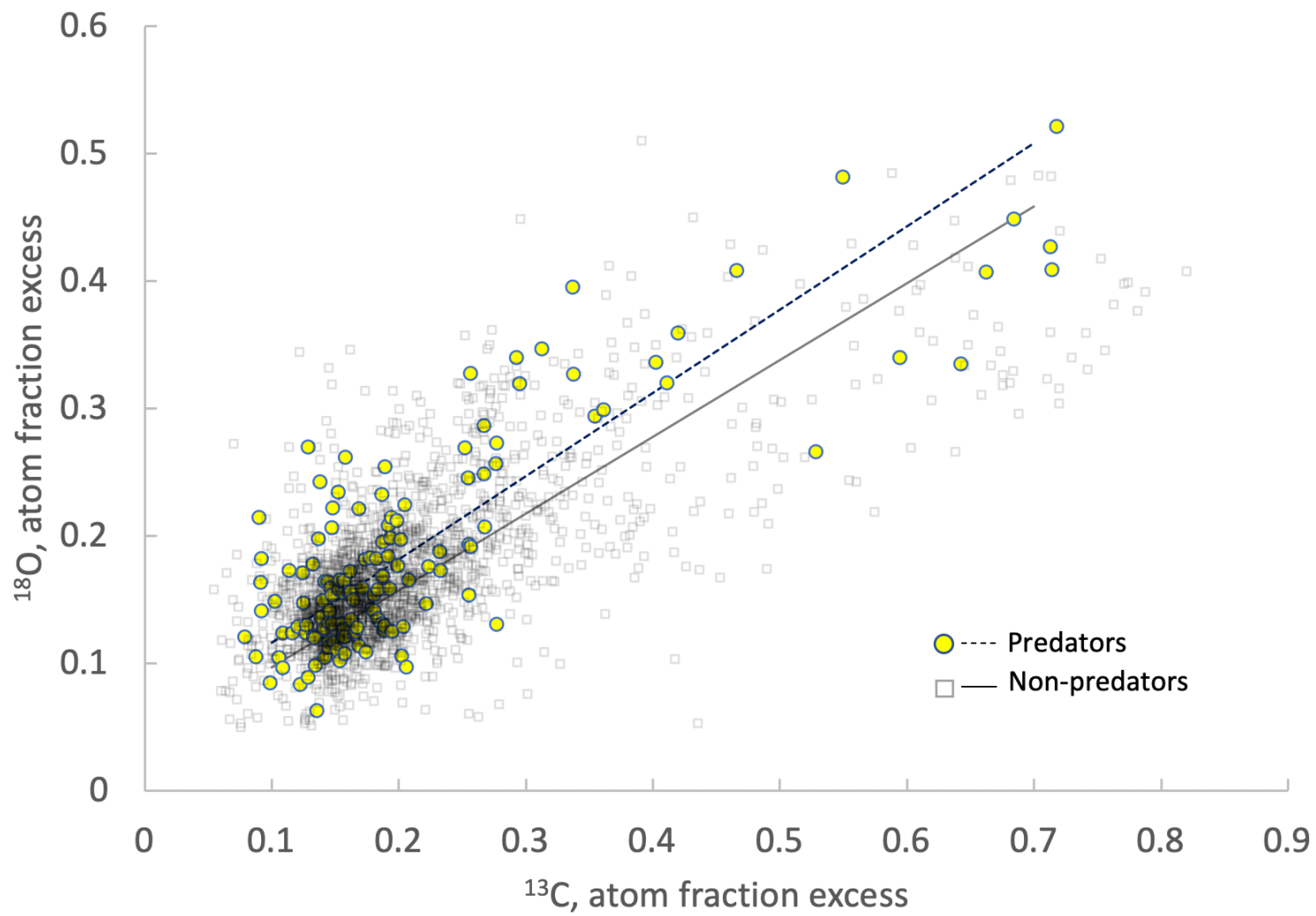

Supplement: FIG S1 [file mBio.00466-21-sf001.pdf]
